# Microbial Nitrogen Cycling in Antarctic Soils

**DOI:** 10.3390/microorganisms8091442

**Published:** 2020-09-21

**Authors:** Max Ortiz, Jason Bosch, Clément Coclet, Jenny Johnson, Pedro Lebre, Adeola Salawu-Rotimi, Surendra Vikram, Thulani Makhalanyane, Don Cowan

**Affiliations:** Centre for Microbial Ecology and Genomics, Department of Biochemistry, Genetics and Microbiology, University of Pretoria, Pretoria 0002, South Africa; emaxortiz@gmail.com (M.O.); jason.bosch@up.ac.za (J.B.); clement.coclet@gmail.com (C.C.); jennyjohnson865@gmail.com (J.J.); pedro.lebre@up.ac.za (P.L.); talk2adey100@gmail.com (A.S.-R.); surendravikram1@gmail.com (S.V.); thulani.makhalanyane@up.ac.za (T.M.)

**Keywords:** N-cycling, soils, Antarctic, diazotrophy, anammox, ecosystem services, bacteria, archaea, Cyanobacteria

## Abstract

The Antarctic continent is widely considered to be one of the most hostile biological habitats on Earth. Despite extreme environmental conditions, the ice-free areas of the continent, which constitute some 0.44% of the total continental land area, harbour substantial and diverse communities of macro-organisms and especially microorganisms, particularly in the more “hospitable” maritime regions. In the more extreme non-maritime regions, exemplified by the McMurdo Dry Valleys of South Victoria Land, nutrient cycling and ecosystem servicing processes in soils are largely driven by microbial communities. Nitrogen turnover is a cornerstone of ecosystem servicing. In Antarctic continental soils, specifically those lacking macrophytes, cold-active free-living diazotrophic microorganisms, particularly Cyanobacteria, are keystone taxa. The diazotrophs are complemented by heterotrophic bacterial and archaeal taxa which show the genetic capacity to perform elements of the entire N cycle, including nitrification processes such as the anammox reaction. Here, we review the current literature on nitrogen cycling genes, taxa, processes and rates from studies of Antarctic soils. In particular, we highlight the current gaps in our knowledge of the scale and contribution of these processes in south polar soils as critical data to underpin viable predictions of how such processes may alter under the impacts of future climate change.

## 1. Introduction

Continental Antarctica is largely ice-covered, with limited coastal, montane and maritime ice-free areas (approx. 54,000 km^2^, est. 0.44%) [1]. Following the advent of modern microbial phylogenetics over two decades ago, the microbiology of Antarctic terrestrial soils has become the subject of extensive research. This research has led to the discovery that these remote and extreme edaphic habitats harbour microbial populations that are much more phylogenetically and functionally diverse than ever previously recognised [2].

Comprehensive phylogenetic surveys of Antarctic soils and soil-related habitats from most of the dominant ice-free areas of the continent, based on 16S (prokaryotic) and ITS (lower eukaryote) amplicon sequencing data or shotgun metagenome sequences, have now been published. Many of the analyses relate to the South Victoria Land McMurdo Dry Valleys, but more remote edaphic zones such as the Vestfold Hills, Mars Oasis and Robinson Ridge have also been covered [2,3,4,5]. Additionally, more “exotic” soil types, including heated alpine soils, ornithogenic soils and niche soil habitats such as biological soil crusts and hypolithons [6], have been the focus of detailed phylogenetics surveys.

There is a growing worldwide emphasis on large-scale biogeographic studies of soil microbial diversity. While such published studies in Antarctica are currently restricted to sub-regions of the continent (such as the McMurdo Dry Valleys [3] and the Antarctic Peninsula [7]), a number of continent-wide soil microbiology biogeographical surveys are currently underway, at least one of which includes samples recovered by the 20th December 2016–19th March 2017 Antarctic Circumnavigation Expedition (ACE) cruise [8], which accessed soils from a series of remote and rarely visited sub-Antarctic islands.

The Antarctic continent is largely devoid of higher eukaryotes, with the lower latitude regions of the peninsula being the only regions to harbour angiosperms (two species: *Deschampsia antarctica* and *Colobanthus quitensis*) and only highly localized peri-coastal areas impacted by marine mammals and birds. Antarctic soils are dominated by prokaryotes, although some lower eukaryotes (fungi, chlorophytes and microfauna) are widespread and others, such as bryophytes and microalgae, occur locally [9]. In consequence, in much of the continental Antarctic edaphic habitat, ecosystem services, including nitrogen turnover, are thought to be largely performed by prokaryotes.

Most Antarctic soils are considered to be severely oligotrophic and are particularly low in organic nitrogen [4]. Nitrate accumulation, as commonly found in hot desert soils, is less prevalent in Antarctic cold desert soils. Highly localized coastal soils, sites of penguin rookeries and elephant seal colonies, are hugely enriched in uric acid and organic nitrogen [10], while lacustrine cyanobacterial and algal mat biomass is a source of edaphic N input to soils in some of the McMurdo Dry Valleys [3]. Elsewhere, N inputs are probably restricted to trace amounts in snowfall [11] and dinitrogen fixation by diazotrophic microorganisms, particularly Cyanobacteria [12].

While an understanding of the distribution of N-fixing and N-processing organisms in Antarctic soils, and knowledge of the presence or absence of key N-cycling genes, is increasingly comprehensive, quantitative data remain remarkably scarce. The very limited data on N-processing rates in any of the different Antarctic soil habitats make it virtually impossible, at the current state of knowledge, to model the nitrogen turnover contributions to ecosystem services in any part of terrestrial Antarctica, with the consequence that an accurate baseline for future estimations of the effects of continental climate change is equally remote.

**Figure 1:**
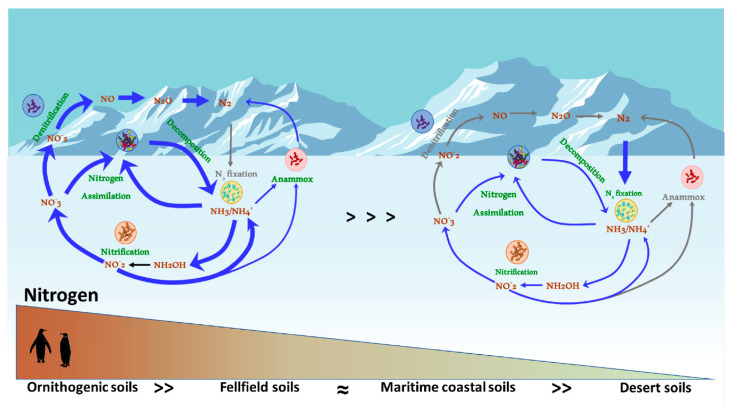
N-cycling processes in Antarctic soils, across the nitrogen hetero-oligotrophy spectrum. Line colour and thickness represent the relative importance of the individual N biotransformation processes in the various soil types.

Here, we review the current state of knowledge of nitrogen processing in Antarctic soils (Figure 1), with an emphasis on the critical gaps in our knowledge base.

## 2. N Compound Speciation and Quantitation in Antarctic Soils

The continental and sub-Antarctic regions encompass a very large latitudinal scale (over 40 degrees of latitude). This geographical scale has led to the delineation of three biogeographic zones, comprising maritime, sub-Antarctic, and continental Antarctica. Maritime Antarctica includes the Scotia Arc archipelagos, South Orkney and South Shetland Islands and the majority of the Antarctic Peninsula southward to Alexander Island. The sub-Antarctic region is represented by various islands close to the Antarctic polar frontal zone. Continental Antarctica includes the eastern and southern regions of the Antarctic Peninsula and typically includes other Antarctic continental territories [13,14].

These biogeographic regions harbour a wide spectrum of varyingly constituted soils. The soils are largely oligotrophic, with widespread stoichiometric imbalances [6]. The availability of total N varies substantially across the different biogeographical zones (Table 1) [15,16,17,18,19,20,21]. These variations in nutrient availability certainly contribute to the phylogenetic and functional differences in microbial communities in soils from different biogeographic regions (e.g., [3]).

Until the recent recognition that understanding the drivers of microbial communities in Antarctic soils requires a systematic quantification of physiochemical variables and biogeochemical cycling, few studies quantified soil chemistry. These studies have, however, demonstrated remarkably low levels of total organic nitrogen (TON) and NH_4_^+^-N in several non-coastal continental soils, although there is evidence of nitrate accumulation in some continental soils [22,23,24]. This excess nitrate is likely to influence soil N stoichiometries and the biological capacity to sequester nitrogen and might ultimately influence the net accumulation of other key elements such as carbon in Antarctic soils. However, due to the sporadic and incomplete data across a range of Antarctic soils, we lack the quantitative estimates required to validate such predictions.

A systematic review of the previous studies shows evidence of low or very low N levels across a range of Antarctic soils. In some regions, N levels are at or below detection levels [25,26]. There have been suggestions that, in regions such as the McMurdo Dry Valleys, the soil N may be derived from nearby coastal and lacustrine algal mats, where katabatic wind episodes transport organic nitrogen from localized high productivity areas to nearby oligotrophic soils [27,28,29]. These studies are supported, in part, by the detection of known cosmopolitan marine Cyanobacteria including *Leptolyngbya*, *Phormidium*, *Oscillatoria* and *Nostoc* in these soils [30,31].

At the opposite end of the scale, ornithogenic soils have high levels of NH_4_^+^-N. Guano deposits from penguins and pinnipeds in large breeding colonies appear to be the primary source of this NH_4_^+^-N, which is derived from the hydrolysis of uric acid [32]. The extent to which diazotrophs in these soils may sequester N is unclear, since the effects of high uric acid concentrations are likely to inhibit N sequestration [32]. Organic nitrogen in coastal soils may be supplemented by marine aerosols or marine algal deposits [33], while nitrogen in sub-Antarctic fellfield soils is probably largely plant-derived [19].

## 3. N-Cycling Taxa in Soils

With the complete absence of higher plants from much of ice-free continental Antarctica, much of the soil nutrient cycling is thought to be driven by microbial communities [34,35] (Table 2). This prediction is corroborated by evidence of the extensive genetic capacity for nitrogen cycling in continental soils and soil-associated niche habitats [34,36]. These functional nitrogen cycling pathways are similar to those previously observed in bacterial and archaeal phyla [34,37]. 

### 3.1. Bacterial Nitrogen Cycling in Soils and Cryptic Niches

As for other terrestrial environments, bacteria dominate Antarctic terrestrial niches [12,26]. Evidence from phylogenetic gene surveys suggests that bacterial taxa mediate the bulk of primary productivity [12]. Cyanobacteria are widely considered to be the central regulators of nitrogen cycling in soils [53]. As direct and indirect mediators of nutrient recycling in Antarctic soils, Cyanobacteria play important functional roles as “ecosystem engineers” [38]. Heterocystous Cyanobacteria, predominantly *Nostoc commune*, appear to drive nitrogen fixation in Antarctic soils. Other heterocystous Cyanobacteria including *Calothrix*, *Dichothrix*, *Nodularia* and *Hydrocoryne* may play a role in sequestering nitrogen in soils and rock-associated niches such as hypoliths and endoliths [39,40,54]. Several studies have reported that nitrogen sequestration genes, linked to rate limiting steps in the cycle including ammonia oxidation, are homologous to those previously found in Cyanobacteria including *Nitrosospira* and *Nitrosomonas* [41,42].

In the McMurdo Dry Valleys, hypoliths and endoliths are important sources of nitrogen in hyperoligotrophic soils [26,36]. In these systems, nitrification is driven by several Cyanobacteria including *Nostoc* and Anabaena [12,43]. Denitrification, the reduction of nitrate to N_2_ gas, is mediated by Deltaproteobacteria, Bacteroidetes [34,36,44] and Actinobacteria [25,37]. Betaproteobacteria and Planctomycetes are also key taxa implicated in soil nitrate removal via the denitrification and anaerobic ammonium oxidation (annamox) pathways [34]. It has been suggested [46] that Burkholderiales, diazotropic Betaproteobacteria, may also play an important role in N input in newly colonized soils. Other taxa, including Actinobacteria (genus *Streptomyces* and family *Frankeniaceae*) and Chloroflexi, are involved in nitrogen fixation in Antarctic soils [44,45], although their quantitative contributions are unknown.

These studies support the conclusion that the capacity for diazotrophy is widespread in nutrient-poor Antarctic soils, other than in certain high altitude edaphic areas [55,56,57]. However, the extent and importance of interactions among Antarctic taxa, and their importance in diazotrophy, is not well understood, although there is some evidence that cooperativity between Cyanobacteria and other taxa (e.g., Actinobacteria, Bacteroidetes and Proteobacteria) is essential for completion of the nitrogen cycle [34,40,58].

### 3.2. Archaea Are Drivers of Nitrification in Antarctic Soils and Niche Habitats

Although the occurrence of Archaea in coastal soils has been well documented [35], they are the least understood members of the microbial community in Antarctic soils. Archaea have been implicated in nitrogen fixation in soils collected from several sites in the Miers Valley [37], while Thaumarchaeota (formerly known as Crenarchaeota Marine Group 1.1b) represents more than 80% of all archaeal sequences in McMurdo Dry Valleys soils [47]. It was suggested that these taxa (collectively known as ammonia oxidizing archaea: AOA) may be the dominant ammonia oxidizers in this edaphic habitat [59]. Thaumarchaeota are known to be key players within the global nitrogen cycle due to their involvement in nitrification across a wide range of habitats [59,60,61] by oxidizing ammonia to nitrite, mediated by ammonia monooxygenase (*amoA*).

Other studies have also demonstrated the presence of AOA, with high abundances of *Nitrososphaerales* lineages, in Antarctic coastal soils [25,41,42,48,49]. Quantification of the relative abundances of AOA and AOB (ammonia oxidizing bacteria) in Antarctic Peninsula soils indicated a general dominance of AOA over AOB [25], but it was noted that the functional importance of AOB vs. AOA could vary in natural ecosystems [62].

Sequences linked to AOA are sporadic in data repositories, with the majority related to Thaumarchaeota previously reported from Antarctic waters and soils [59,60,61]. Overall, a synthesis of the current literature appears to confirm that AOA are ecologically rare in non-maritime Antarctic soils [25,42,63].

### 3.3. The Role of Fungi in Nitrogen Cycling

Eukaryotes in Antarctic soils are largely fungal and dominated by relatively few ascomycete taxa [35,64]. Free-living fungi and yeasts are generally of limited abundance [64,65] and are primarily restricted to lithobiont niches [6]. However, as for Archaea, low apparent abundance does not necessarily imply that these organisms are not important in functional processes related to nitrogen cycling. For example, fungi in Miers Valley soils have been shown to contain nitrification pathway genes [37]. Many fungi, including yeasts commonly found in Antarctic habitats such as *Rhodotorula muscorum*, *Rhodotorula mucilaginosa*, *Cryptococcus aerius* and *Cryptococcus albidus* [50], produce enzymes such urease [66] and may play an essential role in nitrogen mineralization or ammonification. These species can also assimilate many inorganic and organic nitrogenous compounds and are considered to play an important role in nitrogen turnover in soils, including in nitrogen storage [50,51,66]. In addition, although denitrification is generally considered as a prokaryotic process, fungal denitrifiers, identified as *Candida sp* and *Trichosporon cutaneum*, have been found in Antarctic soils [52]. However, to date, the role of fungi and yeasts in nitrogen cycling in Antarctic soils is still poorly understood.

### 3.4. Viruses as Drivers of Nitrogen Cycling

While the composition of some Antarctic soil microbial communities is now generally well understood, the role of associated phages and viruses, and their potential influences on microbial dynamics and nitrogen cycling via host cell lysis, remains poorly understood. It has been speculated that viruses may play a very significant role in biogeochemical cycling of Antarctic soils, particularly by inducing species diversification and consequent functionality [67]. Recent investigations suggest that viruses in Antarctic soils and hypoliths are highly diverse, mainly dominated by *Mycobacterium* phages [68,69]. Studies in other systems have hinted at the importance of viruses in metabolic control [70,71]. It is tempting to speculate that the extreme environmental conditions may promote a lysogenic rather than lytic phage lifestyle, and there is circumstantial evidence of this from Antarctic “metaviromic” studies [69]. While processes such as phage infection-driven niche differentiation may play important roles in modulating nitrogen cycling in Antarctic soils, we lack the corroborating evidence from viral–host interaction studies to confirm this speculation.

## 4. N-Cycling Genes in Soils

Most of the surveys of N-cycling functional markers in Antarctic soils (summarised in Figure 1) have focused on the core genes involved in N-fixation (*nifH*), nitrification (*amoA*) and denitrification processes (*narG* for nitrate reduction, *nirK* and *nirS* for nitrite reduction, *norB* for nitric oxide reduction and *nosZ* for nitrous oxide reduction). Shotgun metagenomic and amplicon sequencing approaches have been used to explore the presence/absence and diversity of key nitrogen cycling genes [72,73], while gene abundances have been monitored using qPCR and its variations [25,42,74,75,76] and Geochip microarray technologies [34,68,77].

*N-fixation*: Diazotrophic members of microbial communities encoding the nitrogenase enzymatic complex are responsible for the biological fixation of atmospheric N_2_ [78]. This key, high energy-cost and irreversible reaction involves the reduction of N_2_ into NH_4_^+^ by the canonical Mo-nitrogenase [40,79]. A functional and mature nitrogenase enzymatic complex can involve multiple genes encoded on different operons [79,80], with a minimal conserved core of six structural and cofactor biosynthetic genes [81]. The canonical nitrogenase contains the electron transfer Fe subunit, referred to as nitrogenase reductase and encoded by the *nifH* gene, and the MoFe protein, a heterodimer containing the active site encoded by the *nifDK* gene [82,83].

*Nif* genes, particularly *nifH*, are highly conserved and present in a considerable number of phylogenetically divergent bacteria and archaea [84,85]. Most studies take advantage of the robustness of the *nifH* gene as a functional marker to identify diazotroph diversity in natural environments [86]. Across the Antarctic region, *nifH* gene analysis has been used to determine the abundance of autotrophic Cyanobacteria, particularly in cryptic soil habitats such as hypoliths and endoliths [34,36,40,68]. However, the presence of Cyanobacteria is not an absolute indicator of N-fixation as there are reported examples of loss of the *nif*H gene from their genomes [87]. However, strong *NifH* signatures assigned to heterotrophic N-fixers are now considered as evidence for large non-phototroph related inputs of nitrogen into oligotrophic Antarctic soils [34,68,75,88]. Diversity analysis of *nifH* markers in McMurdo Dry Valley hypolithic communities indicated that all potential diazotrophs were associated with Proteobacteria taxa [88]. Similar results, at the functional level, showed the presence of heterotrophic diazotrophs in association with Cyanobacteria, where over 50% of total nitrogen fixation was assigned to non-autotrophic taxa [75].

*Nitrification*: Nitrification represents the oxidative portion of the nitrogen (N) cycle, the two-step process whereby ammonia is oxidized to nitrite and subsequently to nitrate [42]. Although most of the surveys of microbial N processes in Antarctic soils have focused on N-fixation, several studies of the diversity and abundance of nitrifiers have been conducted in different regions of Antarctica, including lakes in the Ross Sea region [42,89,90], on the Antarctic Peninsula [91,92] and on McMurdo Dry Valley soils [34,63,93].

Nitrification processes, carried out by AOA and AOB, are restricted to a limited range of taxa [42,63]. Magalhães et al. identified only four AOA and three AOB amoA OTUs in four different and highly heterogeneous Dry Valley soils [42]. These were derived from *Nitrosospira*-like taxa, typically associated with pristine environments and low soil NH_4_-N levels [42,94,95,96,97]. While several studies have shown a predominance of AOA over AOB [61], the ratio of the two clades varies for different Antarctic soils. In the Antarctic Peninsula, archaeal *amoA* genes were dominant compared to their bacterial counterparts [25], but large variations in AOA and AOB *amoA* gene abundance were detected in four Dry Valley soils [42]. It was concluded that soil geochemical properties (i.e., pH, C/N, Mg, Cr, Mn, Co, Ni and Cu) and other environmental variables such as water availability have a significant impact on the relative abundances of AOA and AOB *amoA* genes [42,63,93].

*Denitrification*: To complete the cycle and return the N_2_ to the atmosphere, nitrate reductase, encoded by the *narGHJI* operon, is responsible for the reduction of nitrate to nitrite [98]. In the second step, two types of nitrite reductase catalyse the reduction of nitrite to nitric oxide: a cytochrome *cd*1 encoded by *nirS* or a Cu-containing enzyme encoded by *nirK* [99,100]. Subsequently, nitric oxide is reduced by the nitrite oxide reductase encoded by *norB*, which produces nitrous oxide, a powerful greenhouse product with significant implications for global warming. Finally, nitrous oxide is reduced to N_2_ by nitrous oxide reductase encoded by *nosZ* [101].

In general, Antarctic soils harbour the genetic capacity to complete the denitrification process [6]. The genes responsible for denitrification have been detected in widely different Antarctic soil habitats, including the sub-Antarctic, maritime Antarctica and desert soils and lithic niches in the McMurdo Dry Valleys [25,34,68,74]. However, the abundance and diversity of denitrification functional markers can differ significantly between sampling locations and can be affected by temperature, vegetation type and macrofauna [25,74,102].

## 5. N-Cycling in Antarctic Soils: Rates, Processes and Ecosystem Services

Microbial communities in Antarctic terrestrial habitats are thought to be primary ecosystem service providers through the input of nitrogen gas into the biosphere via nitrogen fixation into ammonia and subsequent nitrification [40]. This assumption is partly informed by the abundance of N_2_-fixing nitrifying microorganisms in both open soils and cryptic niches, such as diazotrophic Cyanobacteria and Proteobacteria [73,88,103], as well as ammonia oxidizing mosses that dominate several Antarctic landscapes [41]. However, only a limited number of studies have focused on the kinetics of the nitrogen cycle within these terrestrial systems (Table 3).

Studies based on the acetylene reduction assay, which measures *nif*H activity of N_2_-fixing microbes [110], have shown that moss-associated Cyanobacteria *Nostoc commune* in the slopes of Vestfold Hills, Eastern Antarctica, contributed between 52 and 119 mg N m^−2^ yr^−1^ to the local ecosystem [104], while cyanobacteria-dominated hypoliths nearby the McMurdo Station are estimated to contribute a combined 0.38 kg N yr^−1^ [36]. Similarly, studies measuring the rates of ammonia nitrification provided evidence for the nitrification potential of soils from geothermally distinct Antarctic Dry Valley [93,108]. While the inconsistency in rate measurement units between studies make them difficult to compare, the consensus is that these rates are biologically relevant for the ecosystem services of Antarctic terrestrial systems [36,93]. In addition to the innate ability of soil microbial communities to fix N_2_, the N-cycle kinetics of Antarctic terrestrial systems is also affected by external inputs such as water incursions from lakes and ornithogenic sources [111,112]. For instance, the wet deposition of guano in the Penguin Colony, Ardley Island, was shown to increase the nitrate input into the soils, which in turn had a negative effect on N_2_-fixation rates while driving N loss through the increase in denitrification rates [102]. Glacial melt streams in the McMurdo Valley have been also shown to contribute to the N input in downstream soil microbial mats through the transport of *Nostoc*-derived mineralized ammonia and nitrate [113]. Another study focusing on ephemerally wetted soils showed that hyporheic sites close to the margins of streams in the Miers Valley exhibited much higher N_2_ fixation rates (0.04 to 5.8 nmol N cm^−3^ h^−1^) than arid sites in the same region, thus demonstrating that the availability of water plays an important role in N-cycling rates of Antarctic soil communities [75]. As transcriptomics and metagenomics data on Antarctic terrestrial microbial communities become increasingly available, more studies need to be conducted to link the stoichiometry of N-cycle gene presence and expression to actual kinetics of ecosystem services in these habitats.

## 6. N Supplementation Experiments

Given the extreme environmental conditions prevailing in terrestrial Antarctic habitats, the indigenous microbial communities may be particularly vulnerable to rapid changes in microenvironment, including nutrient status [114,115,116]. Various in situ and ex situ microcosm studies have addressed the effects of N supplementation on microbial community composition; the results are frequently contradictory, their interpretation is often made more complex by the inclusion of co-variables (temperature, C supplementation) and there is little consistency between studies in the analytical methods.

Nitrogen addition to McMurdo Dry Valley soils, in the form of glycine and NH_4_Cl, induced increases in microbial enzymatic activities, respiration rates and ELFA (ester linked fatty acid) titres [108,117,118,119]. Garwood Valley soils, supplemented with NH_4_Cl, showed a rapid increase in respiration rates (cf. glucose supplemented soils), suggesting that the soil microbial community was N limited [118]. A study of the combined effect of warming and tryptic soy broth supplementation in maritime Antarctica soils on microbial community composition concluded that nitrogen addition, but not temperature, could induce short-term changes in community structure [120]. More specifically, the addition of NH_4_Cl, along with warming in open top chambers, resulted in a reduction in Gram-positive bacterial markers in EFLA analyses [119]. 

Warmer temperatures increase the availability of meltwater from permafrost and glaciers, affecting N-cycling by soil microbial communities [116,121]. For instance, warm and wet conditions stimulated microbial activity when glycine and tryptic soy broth were added to a maritime Antarctic soil in the Signy Island [122], resulting in the increase in both organic and inorganic N. In particular, the input of carbon in Antarctic soils (either through meltwater or other sources) has been shown to significantly change the kinetics of N-cycling, resulting in net N immobilization [117,118] and changes in the N mineralization process [108,118,119,122,123]. Meltwater pulses with C and N addition induced a positive nutrient cycling response in McMurdo Dry Valley soils [116], but increased water and organic substrate availability led to a loss of prokaryotic taxonomic diversity [115,124,125].

Despite the observed changes in the microbial community and functional responses to N supplementation, there is still no clear consensus on the impact of N addition, at any level. There is a clear need for further investigations of microbial community dynamics, coupled with gene expression studies using metatranscriptomics and metabolomics, in order to better understand the resilience and responsiveness of N-cycling taxa in these Antarctic soils.

## 7. Gaps in Current Knowledge

One of the gaps that has become apparent during this review of the current literature is that the data on nitrogen compound speciation and quantification, while well distributed over the different Antarctic soil environments, are old, having been mostly acquired several decades ago. There are questions as to how relevant these data still are as nitrogen availability may have changed in the intervening years [126,127].

Data on both the nitrogen cycling taxa and genes, largely derived from the application of modern next generation sequencing approaches, are abundant and well distributed over the ice-free soils of Antarctica. However, there are still important questions which remain unanswered. Studies of Antarctic soil microbial composition, outside of manipulation experiments such as the addition of water [128], have been performed at single time points. Such experiments do not permit valid conclusions on how the microbial communities change over time. Previous research has shown that Antarctic microbial communities are sensitive to changes in climatic conditions [129,130], so repeated observations will be important to understand how Antarctic soil microbial communities respond to a changing climate and how this affects nitrogen cycling.

Transcriptomic data from Antarctic soils are, generally, sorely lacking, with a few notable exceptions (e.g., [125]). Database searches of EMBL-EBI MGnify, and transcriptome BioProjects in NCBI, using “Antarctica soil” as a search term do not identify any current Antarctic metatranscriptome datasets. This is, in part, a result of the well-known (but generally not reported) difficulties associated with RNA extractions from extremely oligotrophic continental Antarctic soils. Knowledge of which genes are present is important but it does not tell us whether the bacteria that possess these genes are active or not. It is clear that more studies need to make use of metatranscriptomics to identify which genes (and pathways and processes) are expressed and at what levels.

In addition, approaches which rely on PCR primers targeted to conserved gene regions may struggle to cope with the diversity of sequences present in Antarctic habitats (unpublished data). This may be exacerbated if Antarctic microorganisms make use of pathways that are rare or absent elsewhere. For example, alternative nitrogenases may be expressed and have equal or better function than the canonical molybdenum nitrogenases. There is evidence that the contribution of alternative nitrogenases has been underestimated [79].

Studies on the rates of nitrogen cycling in Antarctic soils are rare [38] and tend to be limited in scope, focusing on very specific situations and only a few geographical locations. Part of this may be due to suggestions that there is little to no biological nitrogen fixation in Antarctic soils [111], making the question seem unimportant. There is a clear need for a broader view of nitrogen pathways, rates and stoichiometry, and future investigations will benefit from addressing the previously identified limitations.

Published nitrogen supplementation experiments have investigated how community compositions change but there is still a need to identify changes in gene expression. Such experiments are also good opportunities for metabolomic investigations in order to gain a more in-depth understanding of how nitrogen influences microbial community functionality [131,132]. To the authors’ knowledge, there has only been a single reported metabolomic investigation of Antarctic soil microbial communities [133].

Given that there are only limited data on nitrogen cycling processes in Antarctic soils, and given the importance of N-cycling as a core component of ecosystem servicing, we offer the following recommendations:

(1) In agreement with Guerra et al. [134], there is a need to establish a wider international effort for regular, long-term monitoring of different Antarctic environments in order to acquire data which can show temporal shifts in both soil nutrient content and in microbial community composition.
(2) Given that much of the basic data on soil chemistry from terrestrial Antarctica are decades out of date, new datasets are desperately needed if we are to understand the effects of a changing Antarctic climate.
(3) While there are now numerous published metagenomic studies available for various Antarctic regions and habitats, there is a distinct lack of transcriptomic, proteomic and metabolomic data, all of which are required for a deeper understanding of community function and nutrient cycling dynamics.
(4) Investigations into Antarctic microbial community composition and function should be performed with an awareness of the uniqueness of the continent and its biota. A reliance on homology-based molecular screening methods may skew community structure and function data, given the possibility that Antarctic microorganisms may make use of pathways and genes which may be absent or considered inconsequential in other less “extreme” environments.

## Figures and Tables

**Table 1:** Soil nitrogen levels in various Antarctic soils. (* Not determined)

	Inorganic N		Total Organic N	References
	NO_3_^−^-N	NH_4_^+^-N		
	(µg g^−1^)	(µg g^−1^)	(ppm)	
**Ornithogenic Soils**				
Marion Island	0–130	101–664	-	[18,19]
Signy Island	90	1369		
S. Shetland Island	18.8–35	140–400		
Ross Island/20–35 soil water	0	6000		
**Desert Soils**				
Ross Desert	0–120	6–40	-	[15,16,17,18,19]
Ross Desert	0–960	0–2.2		
Pensacola Mountains	<1–1250	<1		
Pensacola Mountains	0.7–6.4	0.3–1.1		
Victoria Valley	2	1	0.003	
Coalsack Bluff	742	0.1	0.024	
Barwick Valley	1	0.8	0.004	
Wheeler Valley	7	ND*	0.024	
**Fellfield Soils**				
Marion Island	0	2	-	[18,19]
Signy Island/10–20 soil water	1.9–6	43,894		
Signy Island	0.1	0.1		
Coastal Antarctica	43,850	15–20		
**Maritime Coastal Soils**				
Penguin Rock, soils with penguin	31.19	385.52		
Rakusa Point	4.74	66.32		
Puchalski grave, tundra on slope	1.62	23.6	-	[21]
Jersak Hills, scree debris	1.32	1.16		
Arctowski station, base soils	5.25	60.43		

**Table 2:** Summary of microorganisms participating in nitrogen cycling.

Phylum	Taxa	Role	References
Cyanobacteria	*Nostoc commune*	Nitrogen fixation	[38]
	*Dichothrix* *Nodularia* *Hydrocoryne* *Hydrocoryne*	Nitrogen sequestration	[39,40]
	*Nitrosospira* *Nitrosomonas*	Ammonia oxidation	[41,42]
	*Nostoc* *Anabeana*	Nitrification	[12,43]
Actinobacteria	*Streptomyces* *Frankeniaceae*	Nitrogen fixation	[44,45]
	*-*	Denitrification	[25,37]
Bacteroidetes	*-*	Denitrification	[34,36,44]
Proteobacteria	Burkholderiales	Nitrogen input	[46]
	Deltaproteobacteria	Denitrification	[34,36,44]
Chloroflexi	*-*	Denitrification	[44,45]
Thaumarchaeota	Crenarchaeota Marine Group 1.1b	Nitrogen fixation	[47]
	*Nitrososphaerales*	Ammonia oxidizing	[25,41,42,48,49]
Basidiomycota Ascomycota	*Rhodotorula muscorum* *Rhodotorula mucilaginosa* *Cryptococcus aerius* *Cryptococcus albidus*	Nitrogen mineralization, nitrogen assimilation and ammonification	[50,51]
	*Trichosporon cutaneum*	Denitrification	[52]
	*Candida* sp.	Denitrification	[52]

**Table 3:** Quantification of N_2_-fixation and nitrification processes in terrestrial Antarctic soil habitats.

Source	N_2_-Fixation/Nitrification Rates	Year	References
Moss-associated *Nostoc* in Vestfold Hills	52–119 mg N m^−2^ yr^−1^	1983	[104]
Moss community in East Ongul Island	0–15 mg N m^−2^ day^−1^	1987	[105]
Soil and grassland from Macquarie Island	0–1372 nmol C_2_H_2_ reduction h^−1^ 10 cm^−1^	1992	[106]
Dry turf and wet moss carpets, Signy Island	1–4.9 kg N ha^−1^ yr^−1^	2000	[107]
Soil from Garwood Valley	5–1000 ng NO_2_-N g^−1^ soil week^−1^	2006	[108]
Hypoliths in the McMurdo Valley	0.38 kg N yr^−1^	2011	[36]
Soil samples in Anvers Island	12–59 μmol N m^−2^ h^−1^	2012	[109]
Soils in Miers Valley	0–5.8 nmol N cm^−3^ h^−1^	2012	[75]
Biological soil crusts in Ardley Island	0–3 kg N ha^−1^ yr^−1^	2017	[102]
Soil from Miers Valley and Beacon Valley	0.025–0.07 pmol NH_4_^+^ oxidation g^−1^ day^−1^	2020	[93]
